# Comparison of manual gold standard with 2 automatic body condition scoring systems for dairy cows

**DOI:** 10.3168/jdsc.2025-0851

**Published:** 2025-12-18

**Authors:** Anette Matto, Katri Ling, Hanno Jaakson, Siim Teder, Priit Karis

**Affiliations:** Chair of Animal Nutrition, Institute of Veterinary Medicine and Animal Sciences, Estonian University of Life Sciences, 51006 Tartu, Estonia

## Abstract

This study assessed the agreement between manual and automatic body condition scoring systems, with a focus on evaluating 2 automated scoring systems by DeLaval (older and newer) for commercial dairy farms. Manual scoring was conducted by experienced assessors using a standardized protocol, and results were compared with data from automated systems. Manual scores showed strong consistency between assessors, supporting the reliability of the method when properly applied. Both automated systems correlated well with manual scores. However, the older system consistently overestimated scores, particularly in thinner cows, whereas the newer system demonstrated closer alignment with manual assessments under the present conditions, though it slightly underestimated early-lactation body condition loss. Despite some limitations in individual cow assessment, especially during early lactation, the newer system represents a potential advancement and both systems are suitable for group-level management. Further refinement is recommended to enhance its precision for individual animal-level monitoring and research applications.

## Highlights

• Manual BCS showed high consistency across assessors.
• The older automatic BCS system overestimated BCS in thinner cows.
• The newer system's scores seem to be more similar to manual scores.
• Both systems are better suited for group-level BCS tracking.

## Graphical Abstract

**Figure:**
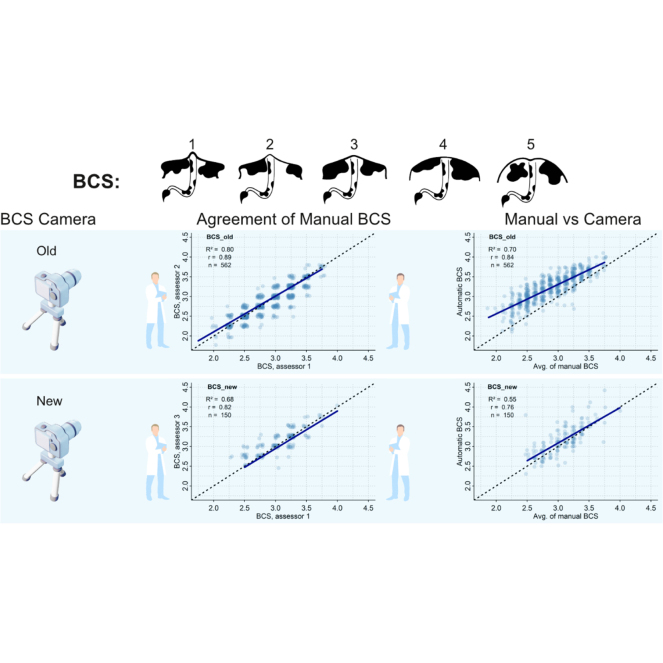
**Summary:** This study compares manual body condition scoring with 2 automated systems developed by DeLaval for dairy cows. Manual body condition scoring, performed by experienced assessors, showed high consistency and reliability. The older automated system tended to overestimate body condition score (BCS), especially in thinner cows, whereas the newer system demonstrated closer alignment with manual assessments under the present conditions. Both systems effectively tracked group-level BCS dynamics throughout lactation, but limitations remain in individual cow assessment, particularly during the critical early-lactation phase. The findings suggest that, although automated systems are suitable for herd-level management, further refinement is needed to enhance precision for individual monitoring and research applications. This study provides valuable insights on the performance of automated BCS technologies and highlights the importance of accurate body condition scoring.

Body condition (**BC**) scoring, a method to monitor the amount and use of body reserves, has been used in dairy cow management for decades. Optimal BCS is necessary to ensure a smooth start to lactation (accompanied by an increase in feed intake), moderate negative energy balance, restart of cyclicity, successful pregnancy, high production level, and preparedness for the next lactation (Roche et al., 2013).

Wildman et al. (1982) and Edmundson et al. (1989) initiated the use of a system with scores from 1 to 5 to determine the BC of dairy cows, independent of body weight and frame size. Principal descriptors of specific body regions in the decision chart of Ferguson et al. (1994) direct the assessor to score a cow between 2.5 and 4.0 (with a precision of 0.25 units), the range most common in cows. The drawbacks of the method are its time-consuming procedure and problems of application, especially in freestall housing systems where cows have more freedom to move around, making it harder to locate, approach, and assess their BC.

Over the years, a great deal of research has been conducted to determine optimal BCS values, for instance, for different lactation stages (Roche et al., 2013), which are applicable to manual BCS. The optimal BCS at calving, based on the 1-to-5 scale proposed by Ferguson et al. (1994), should be 3.25 to 3.50 (Samarütel, 2009) or 3.0 to 3.25 (Roche et al., 2013); the BCS loss after calving is dependent on BCS at calving (Roche et al., 2009; Karis et al., 2021) and should not exceed 0.75 to 1 (Roche et al., 2009).

The dairy industry has changed dramatically since the 1990s. Freestall housing systems on large farms with several hundred cows in milk are the norm in many countries (Barkema et al., 2015; Bewley et al., 2017; Toro-Mujica and Vera-Infanzón, 2024). The application of group feeding on such farms necessitates adequate information on the cows' body reserves and their changes during lactation. Therefore, several methods have been suggested to obtain data on the BC of dairy cows using cameras (Bewley et al., 2008; Halachmi et al., 2013), thermal imaging (Song et al., 2019), or ultrasound (Yukun et al., 2019; Tao et al., 2022), and advanced computer technologies have been proposed to modify the raw data into a BCS (Rodríguez Alvarez et al., 2018; Liu et al., 2020; Zhao et al., 2020).

Since 2015, DeLaval International AB has provided an automatic BC scoring system (DeLaval Body Condition Scoring, DeLaval International AB, Tumba, Sweden) consisting of a 3-dimensional (**3D**) imaging camera coupled with appropriate computer algorithms, which calculate a BCS for the cows passing the sorting gate at every milking (DeLaval, 2015). DeLaval recently implemented a comprehensive upgrade to its BCS system, featuring a new 3D camera (DeLaval, 2025). The first DeLaval automatic BCS system has been compared with manual assessors, but the results have been contradictory. Some have found that the automatic system is suitable for measuring dairy cows' BC for both research and on commercial dairy farms (Albornoz et al., 2022). Others have found it to be poorly specific in certain BCS ranges (<3.00 and >3.75; Mullins et al., 2019).

For automatic scoring to become an integral part of precision farming the producers need assurance that the automatic systems' results are in accord with the manual BC scoring and that the recommended scores are still relevant target values. It is crucial for the producers and researchers to know whether they could trust and correctly interpret the BCS and its change obtained from the automatic system.

The objective of this study was to assess the agreement between manual and automatic BC scoring in Estonian Holstein cows and to compare 2 DeLaval BC scoring systems. To our knowledge, the newer automatic BCS system has not yet been evaluated against manual scoring.

The study was carried out with 2 different DeLaval automatic BCS systems: an older DeLaval BCS (**BCS_old**) and a newer system, DeLaval BCS2 (**BCS_new**). The older system has a Farmic AB (Skellefteå, Sweden) 3D camera and a specific algorithm in the DelPro Farm Manager software (DeLaval International AB), whereas the newer system has a Visionary-T Mini CX 3D camera (Sick AB, Skärholmen, Sweden) with a computer (ARK-1220L-S6A2 Fanless Box PC, Advantech, Taiwan) that uses machine learning to compile and develop the algorithm, and the computed score is sent to DelPro Farm Manager. The BCS systems were installed above the sorting gate of the milking parlor or robot (DeLaval VMS, DeLaval International AB) on 3 freestall farms housing Estonian Holstein cows: the older system on farms A and B (750 and 1,100 dairy cows; average milk yield 11,000 kg and 13,000 kg, respectively), and the newer system on farm C (119 dairy cows and average milk yield 9,900 kg). The systems were adjusted to use the Holstein breed model. Cows were fed a grass silage–based TMR on all farms. On farms with the older system (farms A and B), all cattle were housed in groups based on the stage of lactation and milk production and were milked in a parallel milking parlor. On the farm with the newer system (farm C), cows were divided into 2 milking groups (one milking parlor and one robotic milking group), with grouping decisions made by the manager.

Manual BCS was performed on a scale from 1 to 5 with 0.25-point increments (Ferguson et al., 1994) and was carried out on farm A 5 times from December 2021 to January 2022 with a weekly interval between each assessment. A convenience sample was chosen from fresh, peak, and end-of-lactation groups. On farm B, manual BCS was recorded once in June 2022 with a convenience sample from fresh, peak, and end-of-lactation cows. Lastly, on farm C manual BCS was performed twice, in October and December 2024. On both visits, all lactating cows were scored. The total sample size was based on similar studies (Mullins et al., 2019; O'Leary et al., 2020; Zieltjens, 2020; Albornoz et al., 2022). Manual BCS were given by 2 experienced assessors without communicating with each other. Assessor 1 was the same for all 3 farms. Assessor 2 was the same for farms A and B, but on farm C, experienced assessor 3 was used because assessor 2 was unavailable.

Cows with more than 400 DIM were excluded from the analysis because very few observations were beyond that threshold. Thus, in total, 562 cows' BCS (428 from farm A and 134 from farm B) given by 2 assessors on farms with older DeLaval systems (BCS_old) and 150 (from farm C) with a newer DeLaval system (BCS_new) were matched with automatic BCS and used to compare the manual and automatic BCS. Automatic BCS were obtained from DelPro Farm Manager version 5.1 software for the older system and version 10.3 for the newer system. If the system did not have a score for a cow on the same day as manual scoring, then the cow was recorded with the automatic score closest to but not exceeding 5 d from the manual scoring day (5-d rule). The number of cows without a score on the test day was 38; the 5-d rule was applied for 25 of them, and 12 were excluded from the analysis with the older system. With the newer system, only one cow was excluded.

Statistical analysis was performed with R version 4.4.1 (R Core Team, 2024). The BCS assessment was considered accurate if the absolute deviation was within ± 0.25 for primary agreement and ± 0.50 for secondary tolerance. A Wilcoxon rank sum test from the package “stats” (R Core Team, 2024) was used to compare the scores given by 2 assessors. Spearman correlation coefficients were calculated with a function “cor.test” of the package “stats” to assess the relationship between manual and automatic methods. When comparing the automatic BCS systems with manual scoring, the average BCS of the 2 assessors was used to reduce subjectivity. The average trends of the BCS were visualized with locally weighted smoothing, using the function “loess” of the package “stats.”

The study evaluated the results of 2 automatic DeLaval BCS systems (older and newer) in relation to the average of 2 manual scores. To the best of our knowledge, the newer automatic BCS system has not yet been compared with either manual methods or other automatic systems.

The correlation coefficients between the scores given by 2 assessors (1 and 2) on farms A and B (r = 0.89, *P* < 0.01) and on farm C (1 and 3; r = 0.82, *P* < 0.01) in our study were similar or better than the results of other studies (Samarütel et al., 2001, r = 0.88; Mullins et al., 2019, r = 0.85–0.87; O'Leary et al., 2020, r = 0.67). In an influential study on BCS, Ferguson et al. (1994) compared 4 BCS assessors (3 experienced and one less experienced assessor), the correlation coefficient ranged from 0.763 to 0.858. Although O'Leary et al. (2020) had 2 experienced BCS assessors, the correlation coefficient between them was lower (r = 0.67) than in other studies, possibly due to the use of a different BCS protocol.

In addition to very strong correlations between the results of different assessors, the average scores given by 2 manual assessors in our study did not differ irrespective of the actual pair of assessors (1 vs. 2: 2.86 ± 0.40 [mean ± SD] and 2.88 ± 0.41, *P* = 0.28; 1 vs. 3: 3.04 ± 0.34 and 3.10 ± 0.30, *P* = 0.25). The BCS assigned by manual assessors showed strong agreement across the entire BCS scoring scale. The accuracy of the assessors was also good; 2 assessors gave the same score in most of the cases (1 vs. 2: 56.4%; 1 vs. 3: 52.7%), and virtually all (96.9% vs. 93.3%) scores were the same or differed by no more than 0.25 points (Table 1), the detection limit of the method according to Ferguson et al. (1994). For comparison, the differences between 3 experienced assessors reported by Ferguson et al. (1994) were 0.25 points or less in 90.7% of cases and the BC scoring method was also suitable for use even if assessors were less experienced (Ferguson et al., 1994). Therefore, despite the involvement of different assessors, the consistency of the assigned BCS was excellent. This suggests that when the BC scoring protocol is properly followed, manual scoring is not as subjective a method for assessing body condition as previously suggested by Roche et al. (2009) and Mullins et al. (2019).

**Table 1:** The accuracy (absolute deviation within ±0.25 BCS for primary agreement and ±0.50 for secondary tolerance) of the BCS between manual assessors and manual (MAN) versus automatic (AUTO) systems during the whole lactation and on the first 50 DIM

Item	MAN assessors	MAN vs. AUTO	MAN assessors (DIM ≤50)	MAN vs. AUTO (DIM ≤50)	MAN assessors (DIM >50)	MAN vs. AUTO (DIM >50)
BCS_old						
Sample size	562	562	312	312	250	250
Difference ≤0.25, %	96.9	36.3	96.8	31.4	94.8	42.4
Difference of 0.50, %	3.9	40.4	3.2	40.7	4.8	40.0
Difference >0.50, %	0.2	23.3	0	27.9	0.4	17.6
BCS_new						
Sample size	150	150	32	32	118	118
Difference ≤0.25, %	93.3	76.7	96.9	62.5	92.4	80.5
Difference of 0.50, %	6.7	18.0	3.1	21.9	7.6	16.9
Difference >0.50, %	0	5.3	0	15.6	0	2.5

The correlation coefficients between the average scores of the 2 manual assessors and both the older (BCS_old) and newer (BCS_new) automatic scoring systems were high (r = 0.84 and r = 0.76, respectively; *P* < 0.01) and, for BCS_old, was not affected by farm (Figure 1, A). These are similar or better results than in other studies (Mullins et al., 2019, r = 0.78; Zieltjens, 2020, r = 0.70). Although the correlation coefficient of older system's scores with manual scores was higher than for BCS_new, the scores' primary agreement with the manual BCS was worse compared with BCS_new (Table 1). For example, only 36.3% of the BCS values given by BCS_old were within 0.25 points of the manual score, whereas the comparable value for BCS_new was more than double (76.7%). In addition, the manually obtained average BCS of the studied cows differed from the automatic BCS obtained with the older system (2.87 ± 0.40 vs. 3.21 ± 0.35; *P* < 0.01). On average, the difference was 0.34 points, which falls outside the primary agreement defined in this article. A similar finding was reported by Zieltjens (2020). However, with the newer BCS system the average manual BCS was not different from the automatically obtained BCS (3.07 ± 0.31 vs. 3.15 ± 0.37; *P* = 0.16). The difference between the systems is also illustrated by the alignment of regression lines with the line of unity. For BCS_old, the regression line between manual and automatic BCS clearly deviates from the line of unity (Figure 1A), whereas in the case of BCS_new, they are aligned (Figure 1B). This may indicate that the newer camera system works, on average, more similarly to the manual assessors than the older one.

**Figure 1:**
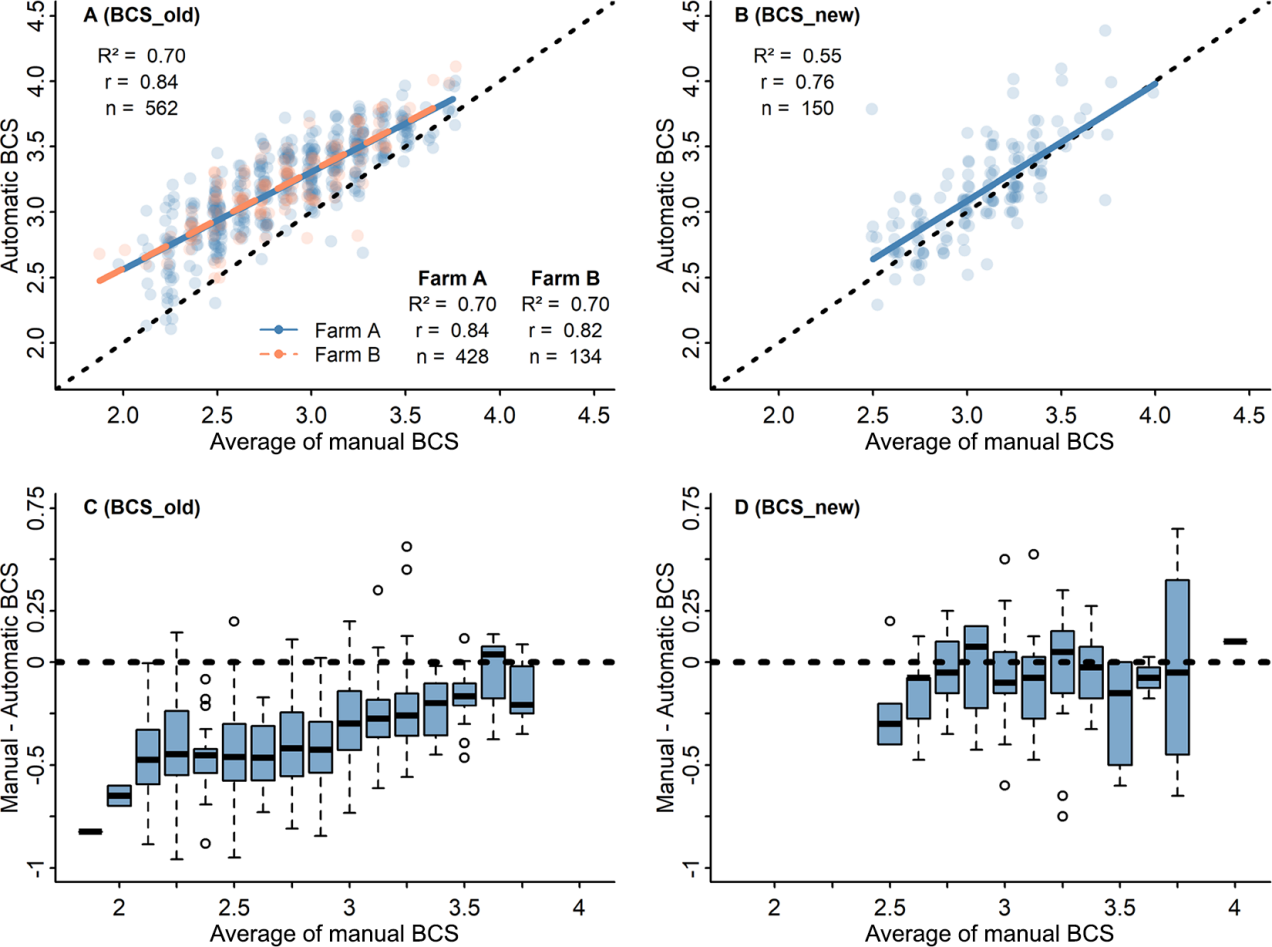
Comparison of manual BCS (average of 2 assessors) with older (BCS_old; n = 562) and newer (BCS_new; n = 150) automatic BCS systems. In panels A and B, each data point represents one assessed cow: its average BCS given by 2 assessors is shown on the x-axis and the respective automatic BCS given by the camera is on the y-axis. The blue solid line indicates the linear trendline between the average of 2 manual assessors and automatic BCS. The boxplots in panels C and D illustrate the difference between the average score of 2 assessors and automatic BCS in relation to average of the manual scores with 2 DeLaval camera systems. Black dotted line indicates line of unity. The blue box represents the interquartile range, the bold black line indicates the median, and the whiskers extend to the minimum and maximum values excluding outliers, which are displayed as open circles.

The regression lines also reveal that the error of the older camera is dependent on the BCS of the cow, as it overestimates thinner cows, which supports previous findings (Zieltjens, 2020). The higher the BCS, the closer the results are between manual and BCS_old; contrarily, as the BCS gets lower, the deviation of scores increases (Figure 1A). For example, if the average manually given score was 3.25, the median BCS_old score was ∼3.5, and if the manual score was 2.5, the median automatic score was ∼3 (Figure 1C). Because BCS_old assessed BC similarly with manual assessors within the range of 3.50 to 3.75 and underestimated thinner cows, the system might not detect deep and prolonged negative energy balance cases, which is an important management aspect of BCS. This could also explain why BCS loss at the beginning of lactation, measured by BCS_old, has a negligible effect on health and production parameters (Pinedo et al., 2022; Hernandez-Gotelli et al., 2023; Hernández-Gotelli et al., 2025). In agreement, Mullins et al. (2019) found that BCS_old gave reliable BCS values within the range of 3.00 to 3.75, but cows with BCS <3.00 and >3.75 were not scored reliably, suggesting that the technology needs adjustments to make it suitable for all body types and to improve its precision and sensitivity, a recommendation also made by Albornoz et al., (2022).

The newer automatic BCS system aligns with the manual scores more closely, the trendline is close to the line of agreement, the difference between manual averages and automatic scores was less than the margin of error throughout the BCS range of 2.75 to 4.0 (Figure 1B and 1D) and there is no systematic overestimation of BCS unlike with BCS_old. Although, on the farm that used the newer BCS system, there were few thin cows compared with the farms where the older system was used. Therefore, we did not get a very good overview of scoring thinner cows with the newer system.

Although BCS_old had some difficulties assessing the individual cow, it describes well the dynamics of the average BCS throughout the lactation (Figure 2). The average BCS of the older system is above the manual assessors' average line due to the reasons laid out previously, but the line's trend itself is almost identical to that of the manual assessors. Meanwhile, with the newer system, the BCS loss at the beginning of lactation is not as deep as described with manually given scores, but from approximately 150th lactation day, the lines are almost identical and describe the dynamics of the average throughout the lactation in the same way. The accuracy of manual BCS with the newer system at the beginning of lactation is worse compared with the whole lactation (62.5% vs. 76.7%, with BCS being the same or differing by no more than 0.25, and 15.6% vs. 5.3%, with BCS differing by more than 0.50; Table 1). Therefore, it can be concluded that both systems are suitable for group-level feeding management, but at the individual-animal level, the older system lacks the necessary accuracy. It can also be discussed whether the newer system is accurate enough to score animals at individual level. As long as automatic systems use manual BCS and its scale as a reference, they should align closely with manual scores to ensure meaningful interpretation. Thus, from both farmers' and researchers' standpoints, it is important to have assessments agreeing with manual BCS, especially at the beginning of lactation, which is one of the most critical phases for dairy cows. Currently, the newer system tends to smooth the BCS loss at the beginning of lactation. In future studies, the accuracy of the new system (both in terms of absolute BCS values and BCS loss) at the beginning of lactation should be further investigated, as the number of cows assessed with the newer system during the first 50 DIM was relatively small (n = 32).

**Figure 2:**
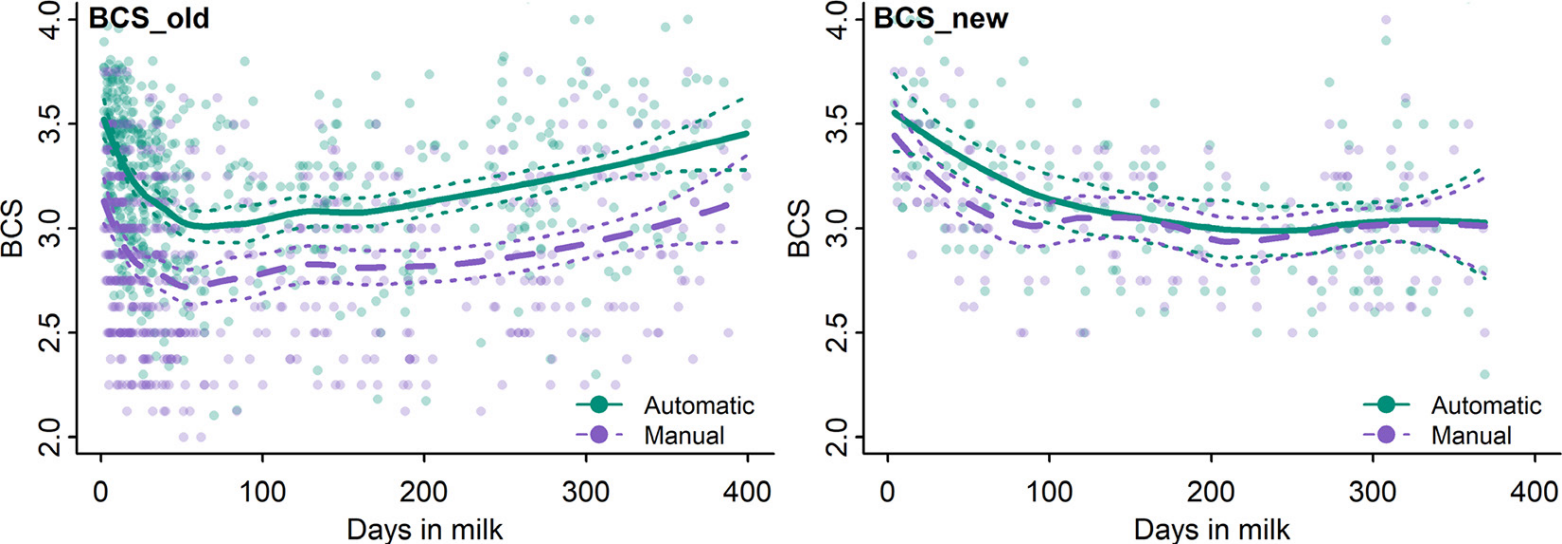
The locally weighted average BCS dynamics up to 400 DIM obtained manually or automatically with 2 different DeLaval BCS camera systems (BCS_old vs. BCS_new). The solid lines represent the average, and the dotted lines represent the 95% CI range.

One limitation of this study is that the systems were compared across different farms, which prevented us from accounting for potential farm-specific effects. Additionally, the newer system had a limited number of observations at the lower end of the BCS scale, which may affect the reliability of conclusions regarding its accuracy in that range.

The manual BCS assessments were strongly correlated, and the average scores given by the 2 assessors did not differ, regardless of which pair of assessors was involved. Thus, manual BC scoring method can be considered reliable, provided that the scoring protocol is correctly followed. DeLaval's automatic BC scoring systems show a strong correlation with manual assessments and effectively track BCS dynamics throughout lactation at the group level. Still, the older system gives systematically higher BCS, overestimates thinner cows and the newer system slightly underestimates the BCS loss at the beginning of lactation. Although the automatic BCS systems have some difficulties assessing the individual cow, the newer system performed better under the conditions of the study. The system requires further refinement to improve its accuracy, particularly for scientific applications and individual animal monitoring at the beginning of lactation. Nevertheless, it is well-suited for use at the group or herd level.
